# Establishing a Wild, Ex Situ Population of a Critically Endangered Shade-Tolerant Rainforest Conifer: A Translocation Experiment

**DOI:** 10.1371/journal.pone.0157559

**Published:** 2016-07-12

**Authors:** Heidi C. Zimmer, Catherine A. Offord, Tony D. Auld, Patrick J. Baker

**Affiliations:** 1 School of Ecosystem and Forest Sciences, University of Melbourne, Richmond, Victoria, Australia; 2 The Australian PlantBank, Royal Botanic Gardens and Domain Trust, The Australian Botanic Garden, Mount Annan, New South Wales, Australia; 3 Office of Environment and Heritage NSW, Hurstville, New South Wales, Australia; 4 Centre for Ecosystem Science, University of New South Wales, Sydney, Australia; INRA - University of Bordeaux, FRANCE

## Abstract

Translocation can reduce extinction risk by increasing population size and geographic range, and is increasingly being used in the management of rare and threatened plant species. A critical determinant of successful plant establishment is light environment. *Wollemia nobilis* (Wollemi pine) is a critically endangered conifer, with a wild population of 83 mature trees and a highly restricted distribution of less than 10 km^2^. We used under-planting to establish a population of *W*. *nobilis* in a new rainforest site. Because its optimal establishment conditions were unknown, we conducted an experimental translocation, planting in a range of different light conditions from deeply shaded to high light gaps. Two years after the experimental translocation, 85% of plants had survived. There were two distinct responses: very high survival (94%) but very low growth, and lower survival (69%) and higher growth, associated with initial plant condition. Overall survival of translocated *W*. *nobilis* was strongly increased in planting sites with higher light, in contrast to previous studies demonstrating long-term survival of wild *W*. *nobilis* juveniles in deep shade. Translocation by under-planting may be useful in establishing new populations of shade-tolerant plant species, not least by utilizing the range of light conditions that occur in forest understories.

## Introduction

Extinction risk for a species is determined by two key traits: population size and geographic range [1]. Translocation, the intentional movement of an organism from one place to another [2], can reduce the risk of extinction in the wild by increasing a species geographic range and population size [3–5]. Translocation is increasingly being used to reduce extinction risk in rare and threatened plants [6,7], with the long-term aim that translocated individuals become established and produce seedlings of their own [6]. However, optimal conditions for establishment of rare and threatened plants are often poorly known [8].

For forest trees, a critical determinant of successful establishment is light environment [9]. The life history strategies of forest trees range from species with seedlings that require high light to survive and grow, and have high maximum growth rates (shade-intolerant species), to species with seedlings that can recruit in deep shade and have slower maximum growth rates (shade-tolerant species) [9,10]. Protection from direct sunlight, as well as the milder temperature and moisture conditions afforded by the understory, is crucial for the successful establishment of shade-tolerant species [11–14]. Under-planting has been primarily developed as a strategy for promoting regeneration of shade-tolerant species that are valued for their timber, such as oak in temperate deciduous forests USA [15,16], spruce in boreal regions in Canada [17] and Sweden [18], shade-tolerant conifer species in coastal temperate rainforest in Canada and the USA [19,20], and for reintroduction of high-value commercial species into degraded forests tropical Americas and Asia [21,22]. The silvicultural theory associated with under-planting for timber production can be applied in species translocation.

Here we report on an experimental translocation (with the aim of informing future translocations, *sensu* [23–25]) of *Wollemia nobilis* (Araucariaceae), a critically endangered, shade-tolerant rainforest conifer [26]. Fewer than 100 mature and 100–200 juveniles of *W*. *nobilis* exist in the wild, in a warm temperate rainforest community in the sandstone canyon landscape of Wollemi National Park in southeastern Australia [27]. Location information for the wild population of *W*. *nobilis* is confidential and protected by law [28]. No genetic variation has been detected in *W*. *nobilis*; genetic analysis revealed no polymorphism at 13 allozyme loci, more than 800 amplified fragment length polymorphism loci or 20 simple sequence repeat loci [29]. Trees 2–20 m tall are scarce, suggesting a serious bottleneck in recruitment from the seedling to mature stage [30]. The viability of *W*. *nobilis* seed is low (~10%; [31]) and seed dispersal is limited—no seedlings have been found >50 m from mature trees. Of those seeds that do germinate, subsequent establishment is also limited, although once established, juveniles can survive in the deep shade for 16 years (and potentially longer) growing very slowly (<2 cm/yr in height) [30]. The optimal conditions for *W*. *nobilis* establishment are unknown [30]. The small wild population and highly restricted distribution of *W*. *nobilis* have contributed to its classification as critically endangered, with the high risk of extinction due to a single event. For this reason, translocation of *W*. *nobilis* has been identified as highly desirable [28]. The understorey of *W*. *nobilis* habitat is characterized by ~3% light, and greenhouse experiments have shown that stem growth of small (<0.7 m tall) *W*. *nobilis* increases with light (up to 50% light; [32]). In particular, we asked:

1. How does growth (stem length, branch number) and survival of translocated *W*. *nobilis* vary along a light gradient?
2. How does the effect of light on *W*. *nobilis* growth and survival vary with plant size?

The terminology used to describe plantings of rare and threatened species for conservation outcomes is often confusing [33–35]. Such plantings have been variously called reintroduction, reinforcement, ecological replacement, assisted colonisation/migration and community construction [33]. Often the distinction between these terms depends on the past distribution of the species. For *W*. *nobilis*, which is only known as living individuals from a single site, but is present in the region (and elsewhere) in the fossil record for hundreds of millions of years [36,37], these definitions are often ill-suited. Throughout this paper, we simply use the relatively broad term “translocation”, which is defined as the intentional movement of an organism from one place to another [2].

## Methods

### Study area

This study was carried out in warm temperate rainforest on land owned and managed by Blue Mountains Botanic Garden Mount Tomah (BMBGMT; -33°32’ S, 150°25’ E). BMBGMT gave permission for the study to be carried out on their land.

### Study design

#### Site assessment for experimental translocation

Potential sites for the experimental translocation were formally assessed nine months before planting. We assessed the suitability of potential sites based on two criteria: (i) habitat characteristics and (ii) logistics, implementation and management [8]. A simplified ranking table was completed for each potential site (S1 Table, *sensu* [8]). The site that we selected for the experimental translocation was in a native warm temperate rainforest community located on land managed by the BMBGMT. The translocation site is in a deeply incised canyon and has environmental conditions that are similar to those of the natural *W*. *nobilis* sites. Mean annual rainfall at the experimental translocation site (1376 mm) was higher than at the wild site (952 mm). We tested soil for N, P, K, micronutrients and pH, to assess similarity to the wild site. In addition, we sampled for the presence of *Phytophthora cinnamomi*, a root fungus that causes dieback in juveniles and adults of many plant species [38] including *W*. *nobilis* [28,39]. Key soil variables at the experimental translocation site were similar to those of the wild site (S2 Table).

#### Light measurements and determination of planting sites (gaps)

We identified 30 potential planting locations in rainforest canopy gaps. We selected the 30 gaps to span the full range of canopy openness at the site. Light was measured from the heavily shaded rainforest (near the creek line) to beneath large canopy gaps at the rainforest-woodland boundary. Light, as a proportion of total available light, at each gap was estimated from hemispherical photography. Hemispherical photography is widely used in studies of canopy structure and transmission of light forest [40]. A camera (Canon 60D camera with a Sigma 4.5 mm hemispherical lens) was mounted on a tripod, levelled and placed so that the top of the photo was facing magnetic north. Three photos were taken for each gap using automatic exposure bracketing (F 14 and ISO 100). Additional photos, one for each plant, were taken at 12 months to capture within-gap light variability, using the same methods. Hemispherical photos were analysed first using the automatic thresholding algorithm in Sidelook [41]. In several instances where the automatic thresholding algorithm did not result in adequate partitioning of canopy and sky, the automatic threshold was noted, and the Gap Light Analysis (GLA 2.0, [40]) software was used to fix problem areas (e.g., sun flare and reflections from rock face). GLA was then used to calculate proportion of canopy and sky and light variables. All analyses were done using the blue channel.

**Fig 1:**
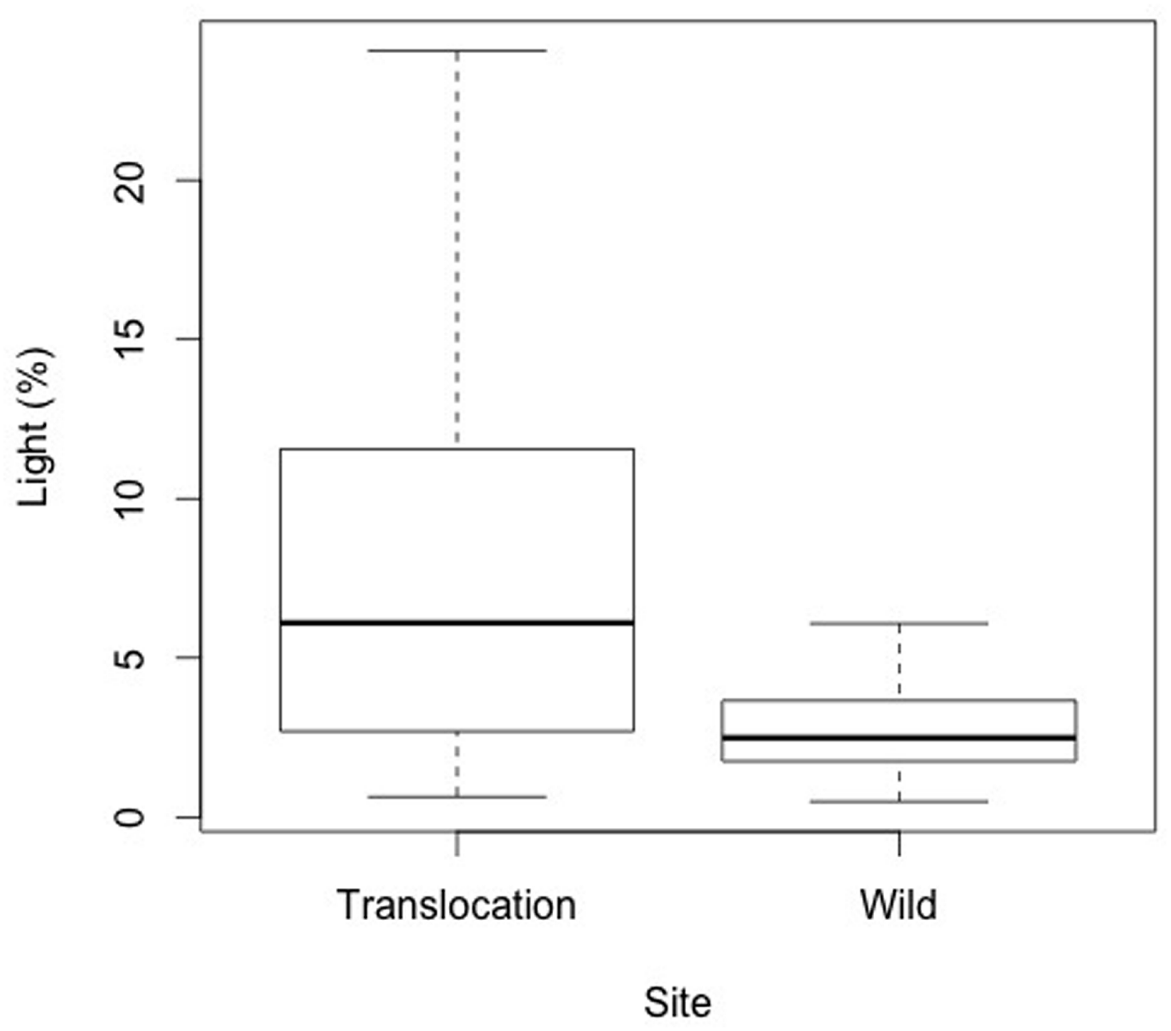
Total transmitted light in the experimental translocation site and the wild *Wollemia nobilis* site.

Transmitted total light at the experimental translocation site ranged from 0 to 24%, compared to the wild site (0–6%, Fig 1; S3 Table). The experimental translocation site therefore provided a greater range of light availabilities.

#### Source material

A total of 191 plants were included in our experiment. One hundred and one plants were obtained from the Australian Botanic Garden Mount Annan (ABGMA), which maintains ex situ populations of *W*. *nobilis*. The plants were 2–3 years old, grown from cuttings from 20 individual wild parents, and were raised in a hygienic environment free from pathogens (e.g., *Phytophthora cinnamomi*). Plants from the ABGMA varied in size. There were 29 plants in 165 mm diameter pots, with a mean stem length of 272 (±13) mm; 55 plants in 250 mm pots, mean stem length 768 (±216) mm; 17 plants in 265 mm pots, mean stem length 1042 (±73) mm. Due to the limited availability of plants from botanic gardens, an additional 90 cutting-grown *W*. *nobilis* were sourced from a commercial supplier. These were in 100 mm pots and had a mean stem length of 456 (±6) mm. Seedlings (rather than plants grown from cuttings) are typically preferred in restoration because of their genetic diversity, and the potential long-term implications for populations [42,43]. However, it should be noted that translocated *W*. *nobilis* populations will have low genetic diversity because the small wild population of *W*. *nobilis* has extremely low genetic diversity [29]. We used plants grown from cuttings rather than seeds or seedlings because of the inadequate availability of seed.

#### Planting

Planting occurred over four days in August 2012 (late winter). Planting was timed to occur before the *W*. *nobilis* growing season (i.e., spring). A quarantine station was established outside of the site, and all equipment was sterilised with a methylated spirits solution to minimize potential *P*. *cinnamomi* spread. Individual plants were manually transported from the road end and placed at planting sites within the warm temperate rainforest. Plants were removed from pots and their roots were gently teased out immediately before planting. In each gap we planted six or seven plants, this included three plants from the commercial supplier (3 × 100 mm pots) and three or four plants from the ABGMA (1 × 165 mm pot, 2–3 × 250–265 mm pots). The experimental translocation site was approximately 120 m long, following a creek, and extending 60 m upslope from the creek (i.e., a total area of 120 m × 60 m). Gaps were spread along the site. Planting was undertaken in an area of approximately 4 m × 4 m within each gap, with at least 1 m between plants. Plant placement was dependent on ability to dig a planting hole (i.e., not impeded by rocks). Each plant was watered (~ five litres), its stem length was measured, and wallaby (and lyrebird) guards were erected. Clearing of ground-level vegetation was sometimes required. In these cases a consistent area (1 m in diameter) was cleared around each plant. Each plant was again given approximately five litres of water one month after planting (September 2012). A total of 191 *W*. *nobilis* were outplanted. Photographs of the plants and experimental translocation site can be found in S1 Fig.

#### Monitoring

Monitoring was undertaken at 1 month (September 2012; survival only), 2 months (October 2012), 5 months (January 2013; survival only), 6 months (February 2013), 11 months (July 2013), 16 months (December 2013), 18 months (February 2014; survival only) and 25 months (September 2014) after planting. Stem length was measured and number of branches counted. *Wollemia nobilis* only produces first-order plagiotrophic shoots (or branches); *W*. *nobilis* branches do not branch [44]. Branches are therefore limited in the size to which they can grow and, in mature trees, they are typically shed after 5–15 years [45]. Signs of insects and pathogens were recorded. The wallaby guards were removed in June 2013 as there were no signs of herbivory on exposed branches and the cages had the potential to restrict branch development as the trees grew.

### Statistical analysis

Preliminary examination of the data showed that plants from the two suppliers had very different trends in growth and survival. Moreover, during planting we observed that all plants from the commercial supplier were root bound and chlorotic relative to the plants from the botanic garden. For this reason, we subset the data by supplier for analysis, and refer to plant origin as either “commercial” or “gardens”.

#### Stem growth and branch number

Total stem growth and number of branches at 25 months was calculated for live plants (*n* = 157; not including those that had died back and then resprouted). We were interested in how growth varied with light availability, and if there were any interactions between light and stem length at t_0_. Stem growth was analyzed according to light, initial stem length (stem length at t_0_) and gap. The same analysis was completed for change in number of branches. We used linear mixed-effects models (lmer in the *lme4* package; [46]) so that a random effect for gap could be included. We fitted a full model: light and stem length at t_0_, their interaction, and the random effect of gap on each, with the form:

$$\begin{array}{l} {\text{Stem growth}}_{ij}\;=\;(\beta_{0}+b_{0j})\;+\;(\beta_{1}+b_{1j}){\text{Light}}_{i}\;+\;(\beta_{2}+b_{3j}){\text{Length}}_{i}\;+\;(\beta_{3}+b_{3j}){\text{Light}}_{i}{\;*\text{ Length}}_{i}\;+\;\varepsilon_{ij}\\ b_{nj}\;\sim \text{ N }(0,\;\sigma^{\text{2}}{}_{nj}\text{)}\\ \varepsilon_{ij}\;\sim \text{ N}(0,\;\sigma^{\text{2}}\text{)} \end{array}$$

where light, length, and their interaction are fixed effects and gap is a random effect. The *β*_*n*_ are the fixed-effects coefficients, which are identical for each gap, and *b*_*nj*_ are the random-effects coefficients, which vary for each gap *j*.

*P*-values were estimated using the Satterthwaite approximation for degrees of freedom in the *lmerTest* package. Models were fit with REML to give unbiased parameter estimates. Effect sizes were estimated using both marginal and conditional R^2^ [47,48] to differentiate between the proportions of variance explained by fixed effects, and the combination of fixed and random effects, respectively.

#### Survival

Plants were classified as alive (*n* = 162) or dead (*n* = 29) at 25 months. Live plants included those that had died back and resprouted (*n* = 6), and dead plants included two plants that had died back, resprouted, and then died (*n* = 2). We were interested in how survival varied with light conditions, and if there were interactions between light and plant size in determining survival, hence survival was analyzed as a function of stem length at t_0_, light, and gap. We used a generalized linear mixed-effects model (GLMM; glmer in the *lme4* package;[46]), as follows:

$$\begin{array}{l} {\text{Survival}}_{ij}\;=\;(\beta_{0}+b_{0j})\;+\;(\beta_{1}+b_{1j}){\text{Light}}_{i}\;+\;(\beta_{2}+b_{3j}){\text{Length}}_{i}\;+\text{ (}\beta_{3}+b_{3j}){\text{Light}}_{i}{\;*\text{ Length}}_{i}\;+\;\varepsilon_{ij}\\ b_{nj}\;\sim \text{ N }(0,\;\sigma^{\text{2}}{}_{nj}\text{)}\\ \varepsilon_{ij}\;\sim \text{ N}(0,\;\sigma^{\text{2}}\text{)} \end{array}$$

where light, length, and their interaction are fixed effects and gap is a random effect. The *β*_*n*_ are the fixed-effects coefficients, which are identical for each gap, and *b*_*nj*_ are the random-effects coefficients, which vary for each gap *j*. To accommodate the binomial structure of the survival data (i.e., dead or alive), a logit link function was used.

Predictor data were centered (mean subtracted) and scaled (divided by two times the standard deviation;[49]). We fit full model as above, although this model, with random intercepts and slopes for both initial stem length and light, was affected by over-specification. For this reason, we present two models, one with a random intercept and slope for stem length, the other with a random intercept and slope for light. Effect sizes were estimated using marginal and conditional R^2^ [47,48]. Predictions for mortality as a function of light were made without the gap random effect (i.e., as GLM), to give an overall picture of the relationship between mortality and light, regardless of gap.

Survival (time to event) analysis was performed using survfit in the *survival* package [50] to investigate patterns in timing of mortality throughout the experiment. The advantage of survival analysis is that it can handle censored observations, where the event of interest (i.e., mortality) did not occur, and incorporate information from both censored and non-censored observations. Commercial and gardens plant data were grouped together in a single analysis. The data were treated as interval censored, as we did not have exact dates of plant mortality. All analyses were undertaken in R [51].

The individual in this manuscript has given written informed consent (as outlined in PLOS consent form) to publish these case details.

## Results

### Stem growth

**Fig 2:**
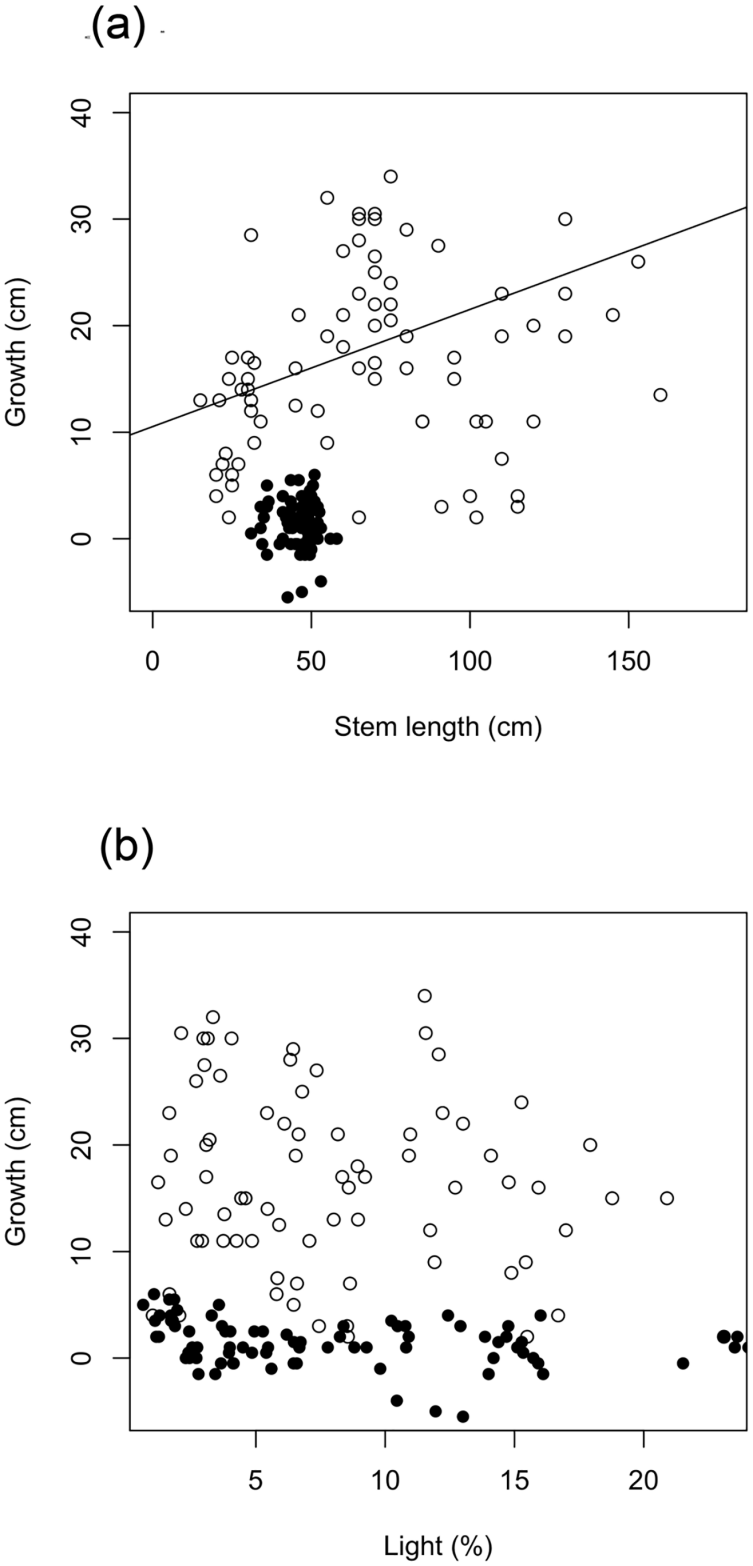
Stem growth as a function of stem length at t_0_ and total light. Commercial plant data are black points. Gardens plant data are white points and solid line. Line represents model estimates (fixed effects) for growth as a function of stem length at t_0_.

The commercial plants grew much less than the gardens plants (Fig 2). Growth over the study period (25 months) was a mean of 14 (±22, 1 standard deviation [SD]) mm for the commercial plants and 160 (±84) mm for the gardens plants, equivalent to annual growth of 7 mm and 77 mm, respectively.

The full model for growth in stem length of gardens plants explained 39% of the variation in the data, 10% of which was explained by fixed effects (marginal R^2^ = 0.10, conditional R^2^ = 0.39; Table 1a). The only significant fixed effect was initial stem length (t-value = 2.337, *P* = 0.026; Table 1a), indicating higher growth in larger plants. The effect of light was not significant. The majority of variation was explained by random effects. The most important random effect was the random intercept for initial stem length, with variance of 2.079 (Table 1a), indicating the effect of initial stem length varied among gaps. However, this was much less than the variance of the residuals of 49.075 (Table 1a).

**Table 1:** Full models of stem growth.

	Fixed effects					Random effects (parameter| gap)			
Parameter	Value (95% CI)	SE	Num DF	*T-*value	*P-*value	Variance	SD	Intercept variance	Intercept SD
**(a) Gardens plants**									
**Intercept**	10.53 (3.82, 17.24)	3.43	52.22	3.075	0.003**	-	-	-	-
**Light**	0.28 (-0.51, 1.08)	0.41	30.56	0.687	0.497	0.020	0.140	0.435	0.659
**Stem length at t**_**0**_	0.11 (0.02, 0.20)	0.05	31.59	2.337	0.026*	0.002	0.043	2.079	0.043
**Interaction**	-0.01 (-0.02, 0.00)	0.01	39.81	-1.228	0.227	-	-	-	-
**Residuals**	-	-	-	-	-	49.075	7.005	-	-
**R**^**2**^	0.10 (marginal)					0.39 (conditional)			
**(b) Commercial plants**									
**Intercept**	3.28 (-4.90, 11.45)	4.17	25.92	0.785	0.439	-	-	-	-
**Light**	-0.11 (-1.02, 0.80)	0.47	33.40	-0.233	0.818	0.002	0.043	0.352	0.593
**Stem length at t**_**0**_	-0.03 (-0.20, 0.15)	0.09	24.74	-0.281	0.781	0.011	0.106	21.972	4.687
**Interaction**	-0.00 (-0.02, 0.020)	0.01	32.76	-0.039	0.969	-	-	-	-
**Residuals**	-	-	-	-	-	4.09	2.021	-	-
**R**^**2**^	0.06 (marginal)					0.16 (conditional)			

* = *P* > 0.05,

** = *P* > 0.01.

In contrast, the model for stem growth of the commercial plants explained only 16% of the variation in the data (marginal R^2^ = 0.06, conditional R^2^ = 0.16; Table 1b). No fixed effects were significant. The most important random effect was the random intercept for stem length, with variance of 21.972 and a standard deviation of 4.687 (Table 1b). This indicates the effect of initial stem length on stem growth varied among gaps.

### Branch number

Gardens plants underwent greater change in branch number, with values ranging from -41 to 25, compared with commercially grown plants, which ranged from -16 to 1.

The model of change in branch number for gardens plants explained 67% of the variation in the data (conditional R^2^ = 0.67; Table 2a), with only 23% explained by fixed effects (marginal R^2^ = 0.23; Table 2a). No fixed effects were significant, although light had a *P*-value of 0.072 (Table 2a). The most important random effect was the random intercept for initial stem length, with variance of 9.712 (Table 2a). However, the residuals had a much larger variance of 62.65 (Table 2a).

**Table 2:** Full models of change in branch number.

	Fixed effects					Random effects (parameter| gap)			
Parameter	Value (95% CI)	SE	Num DF	*t*-value	*P*-value	Variance	SD	Intercept variance	Intercept SD
**(a) Gardens plants**									
**Intercept**	10.34 (2.30, 18.38)	4.10	54.45	2.522	0.015*	-	-	-	-
**Light**	0.88 (-0.06, 1.82)	0.48	60.00	1.829	0.072	<0.001	<0.001	<0.001	<0.001
**Stem length at t**_**0**_	-0.11 (-0.26, 0.03)	0.07	36.45	-1.570	0.125	0.024	0.154	9.712	3.116
**Interaction**	-0.01 (-0.02, 0.01)	0.01	54.81	-1.033	0.306	-	-	-	-
**Residuals**	-	-	-	-	-	62.65	7.915	-	-
**R**^**2**^	0.23 (marginal)					0.67 (conditional)			
**(b) Commercial plants**									
**Intercept**	-4.51 (-19.19, 10.17)	7.49	76.00	-0.602	0.549	-	-		
**Light**	0.93 (-0.75, 2.60)	0.85	76.00	1.087	0.281	<0.001	<0.001	<0.001	<0.001
**Stem length at t**_**0**_	-0.03 (-0.35, 0.28)	0.16	76.00	-0.210	0.831	<0.001	<0.001	<0.001	<0.001
**Interaction**	-0.02 (-0.06, 0.01)	0.02	76.00	-1.242	0.218	-	-	-	-
**Residuals**	**-**	**-**	**-**	**-**	**-**	17.42	4.17	-	-
**R**^**2**^	0.10 (marginal)					0.10 (conditional)			

* = *P* > 0.05.

The full model for change in branch number in commercial plants explained only 10% of the data (marginal R^2^ = 0.10; Table 2b). None of the fixed effects were significant, and the variance within the random effect residuals (variance = 17.42; Table 2b) was greater than any captured by the random slopes or intercepts.

### Survival

The majority of mortality occurred in gardens plants. Ninety-four percent of commercial plants survived, while survival of gardens plants was only 69%. Twenty-nine plants died in total. The native pathogen *Botryosphaeria* infected 17 of the 19 plants that died in <3% light, and 4 of the 10 plants that died in >3% light. *Botryosphaeria* species are opportunistic fungi that predominately affect trees that are injured or stressed [52,53]. *Botryosphaeria* species can be pathogenic to *W*. *nobilis* seedlings [39], but can also exist as non-pathogenic endophytes [54].

**Fig 3:**
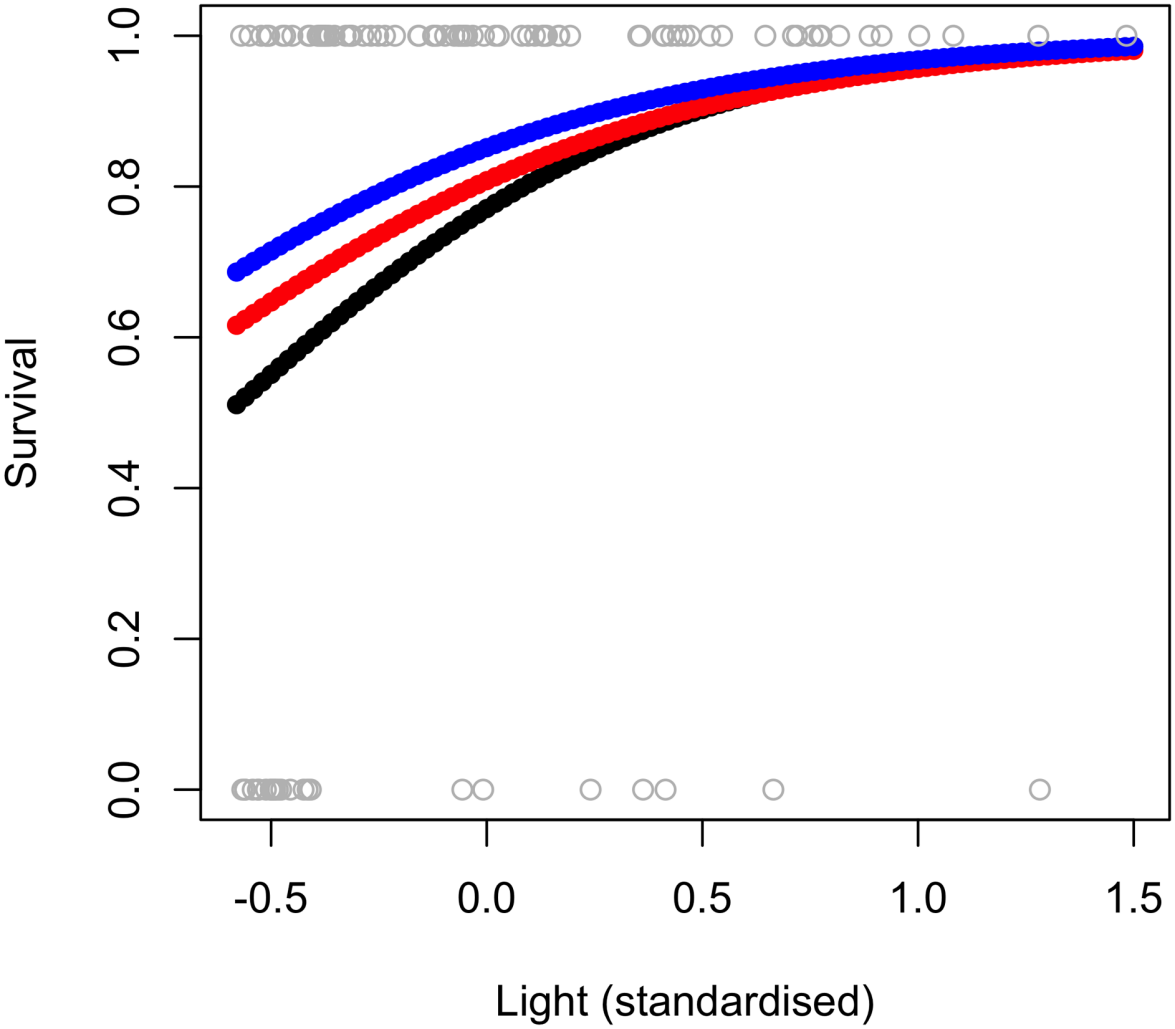
Predicted survival, as a function of light, for plants of a range of stem lengths. 1 = live plant, 0 = dead plant. Predictions based on gardens-supplied plants of a range of sizes: 40 cm (black), 80 cm (red) and 120 cm (blue). Parameter estimates used vary from those in the full model of gardens plant survival (Table 3) as the gap random effect was removed, and the model re-run as a glm to make predictions for all plants, regardless of gap (S4 Table).

The full model of survival of the gardens plants, with a random intercept and slope for light, explained 92% of variation in the data (conditional R^2^ = 0.92; Table 3a). Fixed effects explained 31% of variation (marginal R^2^ = 0.31; Table 3a) and there was a significant positive correlation with light (*P* = 0.037, Table 3a; Fig 3). In terms of random effects, the random slope for light encapsulated more variance (84.433; Table 3a) than did the random intercept for light (5.196; Table 3a). This indicates that the survival response to light varied with gap. The alternative model, with random effects for initial stem length explained only 54% of the data (conditional R^2^ = 0.54; Table 3b). In contrast, for the commercial plants the full model explained only 1% of variation in survival (marginal R^2^ = 0.01, conditional R^2^ = 0.01; Table 4, S2 Fig), indicating that variation in survival of commercial plants was not strongly associated with gap, light or plant size.

**Table 3:** Full models of survival for garden plants.

	Fixed effects				Random effects (parameter| gap)			
Parameter	Value	SE	Z value	Pr *Z*	Variance	SD	Intercept variance	Intercept SD
**(a) Random slope and intercept for light**								
**Intercept**	4.37 (1.21, 7.53)	1.61	2.713	0.007**	-	-	-	-
**Stem length at t**_**0**_	1.79 (-0.14, 3.73)	0.98	1.817	0.069	-	-	-	-
**Light**	7.19 (0.45, 13.94)	3.44	2.091	0.037 *	84.433	9.189	5.196	2.279
**Interaction**	3.20 (-1.59, 7.99)	2.44	1.307	0.191	-	-	-	-
**R**^**2**^	0.31 (marginal)				0.92 (conditional)			
**(b) Random slope and intercept for initial stem length**								
**Intercept**	1.90 (0.63, 3.16)	0.64	2.925	0.003**				
**Stem length at t**_**0**_	-0.34 (-2.28, 1.62)	0.99	-0.336	0.736	1.687	1.299	2.890	1.700
**Light**	1.34 (-0.51, 3.18)	0.94	1.420	0.156				
**Interaction**	-0.79 (-4.04, 2.45)	1.65	-0.478	0.633				
**R**^**2**^	0.07 (marginal)				0.54 (conditional)			

* = *P* > 0.05,

** = *P* > 0.01.

**Table 4:** Full model of survival for commercial plants.

	Fixed effects				Random effects (parameter| gap)			
Parameter	Value	SE	Z value	Pr *Z*	Variance	SD	Intercept variance	Intercept SD
**Intercept**	2.84 (1.93, 3.75)	0.47	6.104	<0.0001***	-	-	-	-
**Stem length at t**_**0**_	0.37 (-1.38, 2.12)	0.90	0.126	0.679	<0.001	<0.001	<0.001	<0.001
**Light**	0.12 (-1.76, 2.00)	0.96	0.414	0.900	<0.001	<0.001	<0.001	<0.001
**Interaction**	-0.22 (-4.48, 4.05)	2.18	-0.099	0.921	-	-	-	-
**R**^**2**^	0.012 (marginal)				0.012 (conditional)			

*** = *P* > 0.001.

Survival analysis (time to event) revealed most of the mortality occurred between the first and second summer, although new mortality occurred at each survey (S1 File).

## Discussion

Translocation is a key strategy to reduce extinction risk for *W*. *nobilis* [28], but the establishment requirements for *W*. *nobilis* were poorly known. While shade tolerance of *W*. *nobilis* had been demonstrated in the wild [30], vigorous growth in high light conditions had been shown in greenhouse studies [32]. We characterised the growth and survival of planted *W*. *nobilis* in a range of light conditions within a new warm temperate rainforest site through experimental translocation. Although it was not our intention, initial plant condition had a strong influence on growth and survival, so our results are considered for each group of plants separately. The commercially grown plants had high survival rates (94%), but also low growth (mean = 14 mm), whereas the plants supplied by the botanic gardens had higher growth (mean = 160 mm) and lower survival (69%).

### Survival

Increased light was associated with increased survival in the gardens-supplied *W*. *nobilis*. Positive relationships between survival and light have been demonstrated in under-planting studies of shade-tolerant species in boreal, temperate and tropical forest types. In Ontario, in east-central Canada, survival of under-planted, shade-tolerant *Picea glauca*, was lowest in uncut (low light) sites, compared to all sites with some level of thinning [55]. Likewise, in the Oregon Coast Range in north-western US, planting seedlings of several conifer species (*Abies grandis*, *Picea sitchensis Tsuga heterophylla*) underneath un-thinned *Psudeostuga menzeiesii* stands was lethal, with all seedlings dead after two years [19]. Alternatively, survival rates increased in thinned stands with higher light [19]. Further, in guidelines for enrichment planting in mixed dipterocarp forest in Sri Lanka, Ashton [56] suggests the optimal conditions for establishment of the majority of species, shade-tolerant and intolerant, are within gaps.

Moving a plant to a new location (i.e., translocation) is likely to influence growth and survival. In our study, the change in light conditions from nursery to experimental translocation site, was likely an important driver of growth and survival responses. Plants alter their photosynthetic apparatus in response to the irradiance that they experience as new leaves form [57,58]. Most of the leaf area of the gardens-supplied *W*. *nobilis* was established in greenhouse conditions of 50% full sunlight. As such, we suspect that higher mortality of gardens plants in darker sites was likely due to insufficient acclimatisation to lower light conditions prior to outplanting. Had the gardens plants been nursery grown in deeper shade, or placed in larger canopy gaps, survival may have been greater. Likewise, survival was enhanced at higher light sites. The ability of shade-tolerant species to respond to increased light availability is well known [59]. Indeed, few tree species recruit well in deep shade and nearly all species increase recruitment and growth with increasing light availability (e.g., [60,61]). For this reason we suggest that future growth and survival will be higher for the *W*. *nobilis* in higher light sites. The relationship between survival and light (in gardens plants) was influenced by gap. We are unsure of what drove this variation, although potential influences include soil variations across the site and, or spatial variation in *Botryosphaeria* infection. Notably neither initial plant size, light nor gap explained variation in mortality of commercially-grown plants.

Most *W*. *nobilis* mortality was associated with infection with a *Botryosphaeria* species (74%, 24 of 29), and occurred in deeply shaded sites characterised by cool and moist conditions. Such conditions are favourable for *Botryosphaeria* growth [62–64]. Fungal diseases are a common problem for threatened conifers globally. In the US and Canada, a key threat to the endangered conifer *Pinus albicaulis* is White pine blister rust (*Cronartium ribicola*). *Cronartium ribicola* is found across the entire distribution of *Pinus albicaulis* and can result in mortality of >50% of infected trees, with mortality more likely in older trees [65,66]. *Torreya taxifolia*, also in North America, is listed as critically endangered because of widespread mortality of adult trees (but not juveniles) caused by infection with *Fusarium torreya* [67,68]. While we had been vigilant about the *Phytophthora cinnamomi* threat to *W*. *nobilis*, the impact of *Botreyosphaeria* was not expected.

Other factors commonly implicated in the survival of translocated plants are herbivory and water availability [69]. There was no evidence of herbivory at the experimental translocation site, and herbivore cages were removed after nine months to allow unimpeded growth. Watering was stopped one month after planting and there was no subsequent observed decline in survival, despite increasing temperatures into summer. The high overall survival of these translocated *W*. *nobilis* indicates that habitat rarity is unlikely to be the cause of the current limited distribution of *W*. *nobilis* (*sensu* [8]), instead disturbance history, or dispersal and establishment limitation (*sensu* [70]) are likely to be more important.

### Growth

Growth of translocated *W*. *nobilis* was not correlated with light, a result that contrasts with greenhouse studies of *W*. *nobilis* [32]. Increasing growth with increasing light is the most typical result reported in under-planting studies from around the world. In temperate US forests, under-planted conifer seedlings, *Thuja plicata*, *Abies grandis* and *Tsuga heterophylla*, had higher growth rates where overstorey retention was lowest [20]. There was also a positive relationship between growth of under-planted (and naturally established) *Pinus strobus* seedlings in the understorey and light in the mesic hemlock hardwood forests of US mid-west [71]. In that study light levels measured at seedlings were not strongly related to gap size, and this was attributed to light interception within the understorey [71]. In a tropical lowland rainforest in Borneo, growth of planted non-pioneer dipterocarp seedlings was higher in gaps relative to seedlings planted in the untreated controls [72]. Similarly, in the Amazon, growth of six under-planted timber species was higher in gaps that were larger, and had higher light (both gap size and light were measured, [73]). Based on these studies we predict that future growth rates of *W*. *nobilis* will be higher in larger gaps. We found that stem growth of gardens plants was significantly correlated with initial plant size—larger plants grew more. The relationship between stem growth and initial plant size was varied with gap, as did the relationship between branch count and initial plant size. Despite early growth of translocated *W*. *nobilis* was not being correlated with light, mean stem growth of the translocated gardens-grown *W*. *nobilis* was much greater than stem growth observed in wild seedlings (77 cf. <10 mm/year; [30]).

The commercially grown plants were in poor condition compared with the garden-supplied plants, showing signs of chlorosis and root binding. Chlorosis can be a response to irradiance [74,75] and/or nutrient status (high or low; [76,77]). Root binding occurs when development of lateral- and/or tap-roots is impeded [78,79]. Restriction of rooting space, regardless of water and nutrient availability, can lead to reduced root growth [80] and whole plant growth after planting [81,82]. While the commercial *W*. *nobilis* had low mortality (i.e., 6% versus 31% in gardens plants), they also grew very little. Variation in the quality of nursery stock can have long-term effects on tree growth and stability [83,84]. In the north-western US, early losses of under-planted conifers (*Thuja plicata)* were attributed to the freezing conditions the seedlings were exposed to before planting [19]. Cole and Newton [85] similarly noted an impact of planting stock quality on the survival of under-planted *Pseudotsuga menziesii* and *Tsuga heterophylla*. Other studies have been established specifically to assess nursery conditions and treatments on outcomes (e.g., *Quercus rubra*, [86]). The chlorosis and root binding observed in commercially grown plants at time of planting may have limited plant growth, but also led to resource conservation and therefore higher survival. Notably, of the 37 *W*. *nobilis* that died back, eight (21%) *W*. *nobilis* subsequently resprouted (although two of these individuals then died). Interestingly, all except one of these resprouting plants were commercially grown.

### Conclusion

Translocation offers the potential to buffer rare and threatened species from extinction by establishing new populations (*sensu* [87,88]). Translocation by under-planting is an approach that utilizes the range of environmental conditions that occur within forest understories, which may be particularly useful in establishing new populations of shade-tolerant plant species.

### Management recommendations

To maximise survival of transplants, future translocations of *W*. *nobilis* should be undertaken within sites with light availabilities at the higher end of the range we tested (>20%), and further work should consider the suitability of even higher light levels. Practitioners may also consider using larger plants to maximize growth.

In this study we used cutting-grown plants because no seed-grown plants were available. Future translocations of *W*. *nobilis* should include seedlings when they become available, to account for the possibility that previously undetected genetic variation may emerge from seeds.

A companion study describing the microbial responses associated with Wollemi pine translocation is described by Rigg et al. [89].

## Supporting Information

S1 Fig Photographs of translocated *Wollemia nobilis* and experimental translocation site. (a) Translocation site showing *Wollemia nobilis* and warm temperate rainforest (Gap 11) (b and c) Translocated *Wollemia nobilis* new growth.
(TIF)

S2 Fig Survival of commercially grown *Wollemia nobilis*, according to height and light. (DOCX)

S1 File Survival analysis. **Fig A.** Survival of translocated *W*. *nobilis* through time. **Table A.** Numerical results of survival analysis showing survival of translocated *W*. *nobilis* through time.
(DOCX)

S1 Table Simplified ranking table *sensu* Maschinski and Haskins 2012. (DOCX)

S2 Table Comparison of soil characteristics at the translocation and wild sites. (DOCX)

S3 Table Gap characteristics. Light conditions and relative elevation of the gaps where *Wollemia nobilis* were planted, and summary survival and growth of *W*. *nobilis* according to supplier and gap.
(DOCX)

S4 Table Parameter estimates for the glm that was used to generate predictions for *Wollemia nobilis* survival according to stem length and light (Fig 3). (DOCX)

S5 Table Wollemi pine translocation data. Includes plant origin, planting site light, survival and growth data.
(XLSX)
