# Supplementary figures and images for: Establishing a Wild, Ex Situ Population of a Critically Endangered Shade-Tolerant Rainforest Conifer: A Translocation Experiment
Source: PLoS One. 2016 Jul 12;11(7):e0157559. doi: 10.1371/journal.pone.0157559 (PMC4942103; doi:10.1371/journal.pone.0157559)

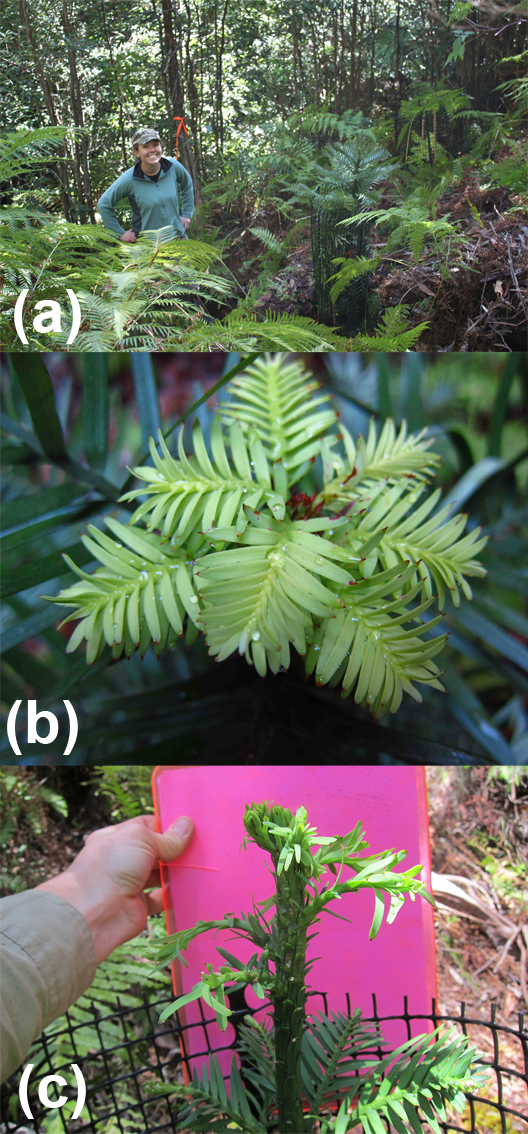

Supplement: S1 Fig — (a) Translocation site showing Wollemia nobilis and warm temperate rainforest (Gap 11) (b and c) Translocated Wollemia nobilis new growth. (TIF) [file pone.0157559.s001.tif]
