# Supplementary material for: Establishing a Wild, Ex Situ Population of a Critically Endangered Shade-Tolerant Rainforest Conifer: A Translocation Experiment
Source: PLoS One. 2016 Jul 12;11(7):e0157559. doi: 10.1371/journal.pone.0157559 (PMC4942103; doi:10.1371/journal.pone.0157559)
Supplement: S2 Fig — (DOCX) [file pone.0157559.s002.docx]

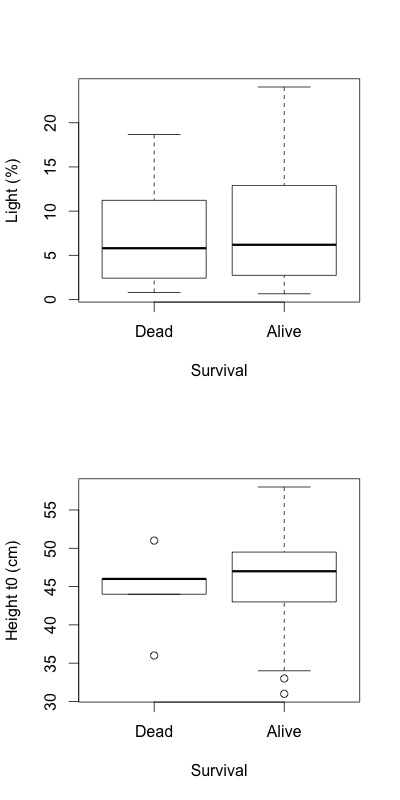


**Supporting Information Figure S2.** Survival of commercially grown plants according to light (above) and stem length (below). There were five dead plants and 85 live plants.
