# Supplementary material for: Establishing a Wild, Ex Situ Population of a Critically Endangered Shade-Tolerant Rainforest Conifer: A Translocation Experiment
Source: PLoS One. 2016 Jul 12;11(7):e0157559. doi: 10.1371/journal.pone.0157559 (PMC4942103; doi:10.1371/journal.pone.0157559)
Supplement: S1 File — Fig A. Survival of translocated W. nobilis through time. Table A. Numerical results of survival analysis showing survival of translocated W. nobilis through time. (DOCX) [file pone.0157559.s003.docx]

**Supporting Information File S1. Table A.** Numerical results of survival analysis showing survival of translocated *W. nobilis* through time.

| Time (months) | Plants (n) | Events (n) | Survival | SE | Lower 95% CI | Upper 95% CI |
| --- | --- | --- | --- | --- | --- | --- |
| 1 | 191 | 1 | 0.995 | 0.00522 | 0.985 | 1 |
| 2 | 190 | 1 | 0.99 | 0.00737 | 0.975 | 1 |
| 6 | 189 | 4 | 0.969 | 0.01262 | 0.944 | 0.994 |
| 11 | 185 | 8 | 0.927 | 0.01886 | 0.89 | 0.964 |
| 12 | 177 | 1 | 0.921 | 0.01946 | 0.884 | 0.96 |
| 16 | 176 | 9 | 0.874 | 0.02398 | 0.829 | 0.923 |
| 18 | 167 | 3 | 0.859 | 0.02521 | 0.811 | 0.909 |
| 25 | 164 | 2 | 0.848 | 0.02597 | 0.799 | 0.901 |


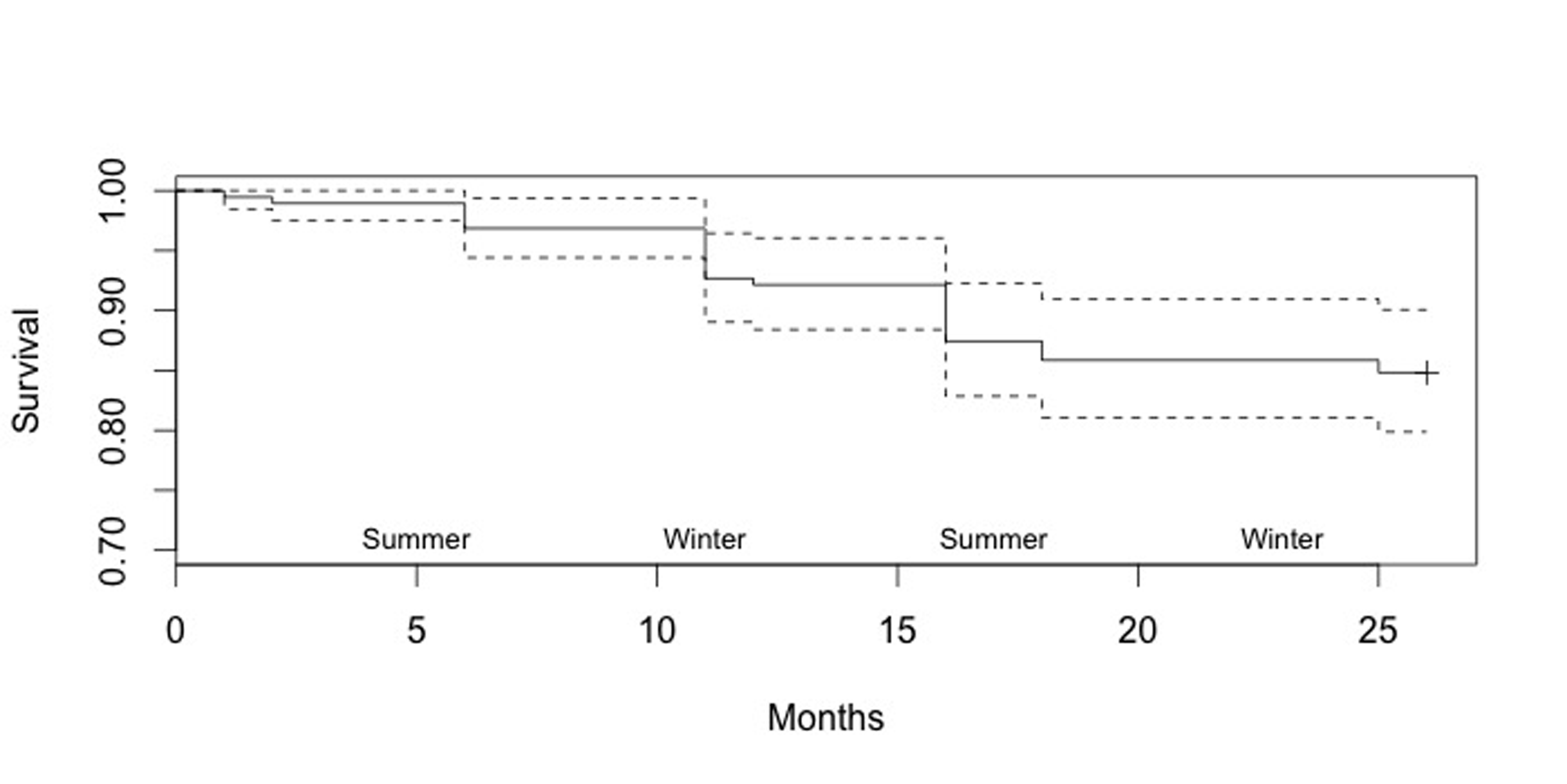


**Supporting Information File S1. Figure A.** Survival analysis showing survival of translocated *W. nobilis* through time.
