# Supplementary material for: Establishing a Wild, Ex Situ Population of a Critically Endangered Shade-Tolerant Rainforest Conifer: A Translocation Experiment
Source: PLoS One. 2016 Jul 12;11(7):e0157559. doi: 10.1371/journal.pone.0157559 (PMC4942103; doi:10.1371/journal.pone.0157559)
Supplement: S1 Table — (DOCX) [file pone.0157559.s004.docx]

**Supporting Information Table S1.** Example ranking table for framework potential sites (*sensu* Maschinski *et al.* 2012).

| **Site:** |  |  |  |  |
| --- | --- | --- | --- | --- |
|  | 3 | 2 | 1 | Score |
| **1. Habitat characteristics** |  |  |  |  |
| - Vegetation similarity | No | Partial | Yes |  |
| - Physiological similarity | No | Partial | Yes |  |
| **2. Logistics, implementation & management** |  |  |  |  |
| - Protection from fire | No | Partial | Yes |  |
| - Water availability | Poor | Fair | Good |  |
| - Public access/human disturbance | High | Med | Low |  |
| - Access for planting and monitoring | Poor | Fair | Good |  |
| - Protection from *Phytophthora* | Poor | Fair | Good |  |
| - Security of land tenure and relationship with landowner | Poor | Fair | Good |  |
| **Total** |  |  |  |  |
