# Supplementary material for: Establishing a Wild, Ex Situ Population of a Critically Endangered Shade-Tolerant Rainforest Conifer: A Translocation Experiment
Source: PLoS One. 2016 Jul 12;11(7):e0157559. doi: 10.1371/journal.pone.0157559 (PMC4942103; doi:10.1371/journal.pone.0157559)
Supplement: S2 Table — (DOCX) [file pone.0157559.s005.docx]

**Supporting Information Table S2.** Comparison of soil characteristics at the translocation and wild sites.

| Unit | Soil character | Translocation site mean (SE) | Wild site mean (SE) |
| --- | --- | --- | --- |
| Mg/kg | Nitrate | 15.1 (9.3) | 8.1 (2.5) |
|  | P | 8.7 (1.8) | 7.3 (4.7) |
|  | S | 6.65 (3.45) | 61.0 (17) |
|  | Fe | 461.5 (23.7) | 227.3 (28.8) |
|  | Mn | 6.0 (1.9) | 149.3 (82.8) |
|  | Zn | 2.9 (0.7) | 13.2 (8) |
|  | Cu | 0.74 (0.08) | 1.9 (0.3) |
| pH | pH in H_2_O | 4.0 (0.1) | 4.5 (0.2) |
|  | pH in CaCl | 3.2 (0.1) | 3.8 (0.2) |
| mS/cm | salinity | 0.1 (0.0) | 0.3 (0.2) |
| Meq % | K | 0.2 | 0.3 |
|  | Mg | 0.7 | 1.8 |
|  | Ca | 0.1 | 4.1 |
