# Supplementary material for: Establishing a Wild, Ex Situ Population of a Critically Endangered Shade-Tolerant Rainforest Conifer: A Translocation Experiment
Source: PLoS One. 2016 Jul 12;11(7):e0157559. doi: 10.1371/journal.pone.0157559 (PMC4942103; doi:10.1371/journal.pone.0157559)
Supplement: S3 Table — Light conditions and relative elevation of the gaps where Wollemia nobilis were planted, and summary survival and growth of W. nobilis according to supplier and gap. (DOCX) [file pone.0157559.s006.docx]

**Supporting Information Table S3.** Light conditions and relative elevation of the gaps where *Wollemia nobilis* were planted, and summary survival and growth of *W. nobilis* according to supplier and gap.

| Gap | Min light | Max light | Elevation * | Gardens  plant survival | Gardens  plant growth | Comm.  plant survival | Comm.  plant growth |
| --- | --- | --- | --- | --- | --- | --- | --- |
| GAP01 | 3.34 | 8.95 | 43 | 100% | 11.7 | 100% | 0.8 |
| GAP02 | 8.93 | 20.90 | 66 | 100% | 7.9 | 100% | 0.6 |
| GAP02SE | 14.69 | 21.52 | 66 | 100% | 7.2 | 67% | 0.4 |
| GAP03 | 6.54 | 21.45 | 43 | 100% | 7.9 | 100% | 0 |
| GAP04 | 8.59 | 16.11 | 39 | 100% | 10.2 | 67% | 0 |
| GAP05 | 8.64 | 23.52 | 29 | 50% | 2.6 | 100% | 0.5 |
| GAP06 | 8.34 | 16.01 | 26 | 100% | 10.8 | 100% | 0.9 |
| GAP07 | 1.51 | 10.78 | 22 | 75% | 9.9 | 100% | 0.6 |
| GAP08 | 13.01 | 17.93 | 46 | 100% | 8.7 | 100% | 0.2 |
| GAP09 | 6.44 | 11.57 | 25 | 100% | 11.6 | 100% | 0.3 |
| GAP10 | 3.43 | 15.29 | 26 | 50% | 5.8 | 100% | 0.2 |
| GAP11 | 1.66 | 12.22 | 45 | 100% | 10.8 | 100% | 0.9 |
| GAP12 | 1.96 | 6.19 | 40 | 75% | 7.4 | 100% | 0.8 |
| GAP13 | 1.01 | 3.57 | 21 | 33% | 1.9 | 100% | 0 |
| GAP14 | 1.65 | 2.01 | 0 | 0% | NA | 100% | 2.3 |
| GAP15 | 2.39 | 9.73 | 20 | 75% | 10.1 | 100% | 0.6 |
| GAP17 | 4.25 | 9.28 | 31 | 100% | 2.6 | 100% | 0 |
| GAP19 | 2.04 | 6.59 | 22 | 100% | 4.5 | 100% | 0.6 |
| GAP20 | 1.06 | 2.41 | 22 | 25% | 9.1 | 67% | 0 |
| GAP21 | 1.65 | 3.74 | 38 | 75% | 7.2 | 100% | 0.6 |
| GAP22 | 1.87 | 5.90 | 7 | 100% | 0 | 100% | 0.5 |
| GAP23 | 2.29 | 6.10 | 57 | 100% | 10.4 | 100% | 0.5 |
| GAP24 | 0.79 | 3.78 | 64 | 25% | 6.5 | 67% | 1.0 |
| GAP25 | 0.64 | 1.86 | 28 | 0% | NA | 100% | 1.7 |
| GAP26 | 1.22 | 5.83 | 50 | 100% | 8.9 | 100% | 0.7 |
| GAP27 | 5.45 | 8.25 | 33 | 100% | 8.0 | 100% | 0.7 |
| GAP28 | 3.29 | 6.73 | 35 | 67% | 6.5 | 67% | 1.3 |
| GAP29 | 8.53 | 15.93 | 27 | 100% | 7.2 | 100% | 0.7 |
| GAP30 | 5.60 | 23.10 | 60 | 75% | 2.1 | 100% | -0.6 |
| GAP31 | 11.74 | 24.05 | 43 | 100% | 5.8 | 100% | -0.3 |

* Relative to lowest elevation in at the translocation site (creekline).
