# Supplementary material for: Establishing a Wild, Ex Situ Population of a Critically Endangered Shade-Tolerant Rainforest Conifer: A Translocation Experiment
Source: PLoS One. 2016 Jul 12;11(7):e0157559. doi: 10.1371/journal.pone.0157559 (PMC4942103; doi:10.1371/journal.pone.0157559)
Supplement: S4 Table — (DOCX) [file pone.0157559.s007.docx]

**Supporting Information Table S5.** Parameter estimates for model which was used to generate predictions for *Wollemia nobilis* survival according to stem length and light in Figure 4.

| Parameter | Value (glm) | St. Dev (glm) | Z (glm) | Pr(z) (glm) |
| --- | --- | --- | --- | --- |
| (Intercept) | 1.323 | 0.272 | 4.863 | <0.001 |
| Stem length at t_0_ | 0.276 | 0.594 | 0.464 | 0.6423 |
| Light | 1.598 | 0.654 | 2.433 | 0.015* |
| Interaction | -1.048 | 1.489 | -0.704 | 0.483 |

We used the same variables as in the best model (for survival of gardens supplied plants, described in Table 3) except the random effect (gap) was removed. We must recalculate without random effects because the change in probability over different values of the predictor (light, height) is dependent on random effect (i.e., group - gap). The effects are dependent on other predictors and group membership. Hence the model is a generalized linear model (glm) rather than a generalized linear mixed model (glmm).
